# 2466: Communication frequency and content between parents of concussed youth and systems of care

**DOI:** 10.1017/cts.2017.281

**Published:** 2018-05-10

**Authors:** Sarah Terry, Molly Cox, Alexandra Linley, Jilian O’Neill, Laura Dreer

## Abstract

OBJECTIVES/SPECIFIC AIMS: To characterize parent communication frequency and content between systems of care (medical, school, and sports/recreation) of concussed youth who are in prolonged recovery. METHODS/STUDY POPULATION: In this ongoing study, 16 concussed youth (average age=14.9 years, SD=1.5; 31.2% female and 68.8% male) and their parent study partner (average age=44.3 years, SD=4.3; 87.3% female and 12.5% male) have been enrolled to date from sports medicine clinics. Demographic information was obtained during the initial clinic intake session. Weekly phone calls were also conducted with the parent and child until the child was considered asymptomatic (ie, reporting no symptoms on the SCAT3), to collect data on communication with the school, sport/recreation, and medical systems throughout the recovery process. For the purpose of this study, we evaluated communication patterns of those parents who had a child in prolonged recovery (ie, symptomatic 14 d or more post-concussion injury). Communication variables included frequency (ie, number of times a parent contacted or attempted to contact a system of care) and content or topic discussed during the contact event. RESULTS/ANTICIPATED RESULTS: Of the 16 enrolled participants to date, 68.8% (n=11) experienced concussion related symptoms 14 days postinjury (M=22.2, SD=4.6) at the time of their 2 week follow-up call and were thus considered to be in prolonged recovery. Of those 11, 81.8% (n=9) of parents reported communicating with the school system at some point between the initial clinic intake session and the 2 week follow-up phone call. The frequency of communication for this period ranged between 0 and 10 instances of contact (M=2.5, SD=2.9). Of the 11 prolonged cases, 8 participants were members of sports teams. Sixty-three percent (n=5) of those parents with a child on a sports team communicated with a coach while none of the parents contacted a team athletic trainer. The frequency of communication with the coach ranged from 0 to 8 (M=1.5, SD=2.5) over the course of 2 weeks from enrollment. With regards to the medical system, the majority of parents (72.7%, n=8) communicated at least once with a medical professional during the same time period. The frequency of communication with the medical system ranged from 0 to 8 (M=2.2, SD=2.6) points of contact. Themes that arose for communicating with the school system included informing school personnel of academic accommodations prescribed by the physician, explaining absences, and concerns about missed academic work and grades. The content of communication with the sports system (ie, coach) pertained to return-to-play issues as well as progress updates on recovery. Themes for communication with the medical system were centered on scheduling appointments, attending follow-up medical appointments, and starting return-to-play protocols. DISCUSSION/SIGNIFICANCE OF IMPACT: Parents of concussed youth who were still in prolonged recovery, for the most part, appear engaged in communicating with multiple systems of care. However, a subset of parents did not participate in contact with these systems. Further discussion of these findings will highlight areas for improvement in concussion management as well as strategies parents can utilize to advocate for their child in terms of return-to-learn and recovery.

